# Casualty Departments

**Published:** 1955-09

**Authors:** Herbert K. Bourns

**Affiliations:** Consultant Surgeon in Charge of Casualty Department, United Bristol Hospitals


### CASUALTY DEPARTMENTS

BY

HERBERT K. BOURNS, M.B., F.R.C.S.

*Consultant Surgeon in Charge of Casualty Department, United Bristol Hospitals*

There have been considerable changes in every branch of medicine since the inception of the Health Act, and in no branch is this more obvious than in the casualty departments of our hospitals throughout the country. There are many reasons for changes and some of the main ones are discussed under three main headings.

Firstly the public have changed in their attitude to the hospitals. The nearby institution is no longer their hospital in which they take a pride and for which they organize collections and fairs. It is now a part of the National Health Service and the doctors and nurses who work there are State employees. Since it is no longer their hospital but a government responsibility they have little respect for it. Since they now have no direct financial responsibility or interest, it is a concern in which the best should always be available, no matter what it costs. Many, of course, believe that the major portion of their weekly contributions under the National Health scheme is directed to the hospitals and doctors. For this reason they do not see why there should be any need for economy in either equipment or personnel. The general public now regard the casualty department as a convenience to be employed at will, and to be criticized freely. Any shortcomings which in their opinion exist, must be corrected regardless of what this may involve in extra staff or money. Judgement is passed hastily and without any consideration of any difficulties with regard to either staff or accommodation.

Secondly the doctors view the casualty department in a different light. There is no point in a doctor under present-day conditions of general practice, carrying out minor surgery or treating extensive septic conditions which require incisions and dressings. He is not repaid either by the State or the patient for his trouble, and he only adds further anxiety and burdens to his already over-full day. The doctor too has to contend with long waiting lists for out-patient appointments. Every patient wants to see a specialist. A case may not be acute but a second opinion may be of little value if it can only be obtained in two months' time. The G.P. adopts the simple expedient of sending such a patient to the casualty department. A second examination is made by a doctor and this is reassuring both to the doctor and the patient. The idea in the mind of the public that a hospital doctor is always better than their own general practitioner still exists and the youthful face of the junior hospital officer does not seem to disturb this faith.

The third factor which is bringing about a great change in casualty services is the large number of industrial accidents which require immediate treatment of a high standard. Minor injuries require expert attention if they are not to have major consequences. The treatment accorded to a lacerated finger in the first twenty-four hours may make or mar the final result. Full function may not be achieved because the primary treatment was inadequate and ill-conceived. With the increase in mechanization and the shortage of skilled workers, the numbers of accidents are greater and the results of inexperienced treatment more disasterous than ever before.

### SERVICE MUST BE ADEQUATE

It is easily discerned, then, that a casualty department is nowadays a place where expert opinion and first-class technique should always be available. Anything less than this is no longer justifiable. To turn to each class of person which utilizes the casualty service of the local hospital, and to lay down strict rules of procedure which must not be transgressed is not to recognize their difficulties and to alienate good will and co-operation. To turn away everyone who cannot justify his presence on all counts is to try to put the clock back, and does not relieve us from the responsibility of producing a much improved service.

Reorganization of casualty departments must be carried out everywhere. Consultants in hospital and doctors in practice must recognize this, but they must also both acknowledge the difficulties during the present period of change, the rate of which is governed by many factors outside the control of medical administrators.

A casualty department must be organized to cope with patients when neither the numbers nor the diseases are known in advance. The notice " House Full" can never be placed over a closed door, but neither should the notice "Welcome" be placed upon the threshold. To cover a busy department so that immediate attention can be accorded to all, means over-staffing unless the number of attendances per hour can be found to follow a rough pattern. In a recent survey at the Bristol Royal Infirmary this was done, and the hourly attendances were noted for a week. It was apparent that attendances did follow a rough pattern with the periods 9.0–10.0 a.m., 10.0–11.0 a.m. and 2.0–3.0 p.m. showing about equal and maximum attendances over all other periods. The introduction of "block" appointment systems for all re-attendances is essential in the future but requires a considerable clerical staff, and accommodation large enough to allow for separation of both new and old cases. Planning will help, but even when numbers are accurately forecast, the type of case which will attend at any time cannot be predicted. One morning may see three serious accidents with the resultant severely injured to be treated. One casualty officer is thus immobilized for a long time and the cases to which he normally attends will inevitably have to wait and so accumulate because they cannot be turned away.

It will be appreciated then that staffing presents a difficult problem, but in another respect the variation in the type of cases may alter the whole demand for medical attention and time on any particular day. Many cases require operative treatment beyond the skill of either nurse or student. To undertake a skin graft, remodel a partially amputated finger or remove a small skin lesion from an important region is a short procedure easily performed under local anaesthetic, when the equipment and the medical staff are available. But a Casualty Officer will put a strain on his own skill and the patient's understanding if he tries to fit these cases into his daily routine. To pass such cases on to the staff of the hospital is uneconomical in both time and beds. The surgical team on duty may be in the middle of a busy out-patient session or a long operating list. The patient cannot be attended to until a convenient moment arrives and this may not occur until many hours have passed. He may even have to be admitted because the operation is not carried out until a late hour. Delays such as these are bad for the patient and his lesion and for the hospital. The casualty services must be equipped for such cases within its own resources.

### JUDGEMENT NEEDED BY CASUALTY STAFF

We have already stated that both patients and, to a lesser degree, doctors use the casualty department because its doors are always open. Many patients plead ignorance of their own doctors' surgery hours. Many state that they cannot wait until the next surgery and again many reply, when questioned about attending the hospital, that they do not wish to bother the doctor with such a trivial matter! All patients have to be examined and assessed before they can be safely sorted out. Many non-urgent cases not referred by doctors occupy valuable time which should be devoted to the treatment of emergencies. Medical officers must be available to deal with these cases because a hospital cannot refuse treatment to any person until his category has been established on competent medical advice. For the general practitioner the despatch of a patient to the casualty department may be the only method by which the patient can be presented quickly to the hospital.

Again, an unknown person collapses in the street or at work. He is taken to the hospital at once. He is directed to the casualty department. He may present a most difficult problem which may be quite outside either the experience or knowledge of a junior medical officer.

A case of multiple injuries is brought in. There may be indefinite signs of both head and abdominal injuries. It requires a doctor of great experience to sort out such a case and to try to balance the contesting demands in diagnosis and treatment.

Who is to deal with all these cases in the hospital?

Will the general practitioner of many years' standing who is worried about a patient be satisfied to have a junior medical officer as a second opinion? Will the hospital authorities and the Minister of Health be content with an organization in which the most junior medical man is faced with the most difficult and the most urgent problems? There are other channels of disposal open to the doctor, such as domiciliary consultation, but these are not always easily available. There are no other methods available to the hospital authorities other than a great increase in the number of consultants and registrars, if the urgent cases are not to be directed to the casualty department.

In staffing a department, therefore, there must be medical staff sufficient not only to deal with the daily routine, but adequate to cope with peak periods and times of unexpected emergencies. This will mean that routine work can carry on quietly and without serious dislocation at times of stress. In addition there must be a medical officer available to carry out the surgical procedures for which all casualty departments must be equipped. Otherwise the junior medical staff will be so pressed for time that both they and their work will suffer, and very soon reach a dangerously low standard. Fatigue will come on earlier and with more serious results. Mental exhaustion is a most important factor. In any business, experts are continually watching the personnel so that their work is balanced and in many cases frequent changes and liberal breaks are given in order to promote efficiency, production and accident-free work. In a Casualty Department, house officers must not be driven so that they become fatigued. There is a continuous demand for acute and keen mental discernment and, in order to maintain a high level of efficiency, liberal time for leisure must be afforded to all the staff.

### NEED FOR SENIOR MEDICAL OFFICER ON DUTY

With regard to the senior medical staff required, it is quite impossible for the hospital to provide cover for serious cases from the resources of visiting specialists and registrars. They have their daily routine which fully occupies their time, and they cannot be expected to run the casualty department at irregular intervals during the day. The examinations of a case of either collapse or serious injury takes at least half an hour, and investigations such as an X-ray examination considerably prolong this period. It is obvious, therefore, that a consultant or registrar or both, must be on the casualty staff. His experience will be invaluable in teaching and supporting the junior house officers. His opinion on subacute cases about which a medical colleague in general practice is worried, will be available and useful. His skill will be present to perform all manner of surgical procedures which many may conceive as minor, but which are now generally recognized as being as worthy of medical skill as an inoperable cancer or an incurable nervous disease. His confidence will be equal to the most difficult and truculent patient who will have to be firmly and correctly treated without medico-legal repercussions of a serious nature. Lastly the presence of a consultant will make for a restoration of confidence in the casualty departments of our hospitals.

The senior man must not only be easily available, but must be present in the department, and for two main reasons. Firstly, when a junior medical officer makes a mistake it is rarely because he is slack but nearly always because, in his inexperience, he does not recognize the seriousness of a case. Secondly, if a second opinion is not readily available to him, he will not seek it as frequently as he should. In large hospitals this consultant cover can and must be provided. In smaller hospitals the consultants or their deputies must be involved, and a return to the rank of Resident Surgical Officer would be a great help. This man can be responsible for and help the Casualty Officer.

### NEED FOR BEDS IN CASUALTY DEPARTMENT

Adequate in-patient accommodation is of great and vital importance. Without beds the casualty department will be severely handicapped. Firstly the beds must be available to and under the direct control of the staff of the department. The rate of admission and discharge is much higher than in any other department of the hospital. There will always be cases of head, abdominal and other injuries which, although not serious, cannot be sent home safely. Observation in hospital will be necessary for at least twenty-four hours. Secondly there are many inflammatory lesions in which a short course of intensive treatment at once will be the most effective therapy if time is not to be wasted in stemming the disease and in repairing its effects. Thirdly patients on whom minor surgical procedures are carried out, may require a bed for recovery, and unless there are beds in the department, it may be impossible to find beds for such cases without a great deal of planning. There are many other reasons why beds are an absolute essential and their provision brings relief not only to the casualty department, but to the whole hospital.

### SEPARATE CASUALTY OPERATING THEATRE

An operating unit is as necessary as in-patient accommodation in any modern casualty department. Small ill-equipped operating theatres have no longer any place in a hospital where casualty and accident cases are received in any numbers. No operation should be undertaken unless full facilities for all that is found to be required in both time and equipment is available Under present conditions many cases have to wait until an operating theatre is empty before essential and urgent surgery can be carried out. This wait is most undesirable. Open wounds, severed arteries and nerves require immediate treatment. The degree of urgency of all the work carried out in a casualty and accident department is much higher than that affecting the rest of the hospital. It is most unsatisfactory that a young fit person with a serious injury should have to compete for theatre time with an old person with a rupture or some other non-urgent surgical problem. Both require treatment but their demands in time are not comparable. An operating theatre in the casualty department adjacent to the wards will get over this difficulty. In it traumatic cases would have priority, and the clinical state of the patient rather than other non-clinical considerations would decide the time of operation. Only then will the advances made by experimental surgeons be brought to bear in the proper manner on cases of trauma. Only then will the advantages of rapid resuscitation be utilized in the most economical and skilful manner. Time must be made to serve the case and not the other way round.

### PROBLEM OF FRACTURES

Cases in which bones are injured attend all casualty departments in large numbers. They are a special problem now that fractures are treated in special units by orthopaedic surgeons. In some hospitals these cases are referred to the fracture department as soon as a lesion is noted and they are taken over by the specialist staff. This at first sight would seem to be satisfactory, but there are two serious objections. In the first place the lesion has to be diagnosed by a casualty officer who has little contact with fractures and no opportunity of increasing his knowledge of the broken parts. He cannot be competent to assess each case as to its seriousness or its urgency; since he does not treat fractures, he does not become experienced in the interpretation of X-rays. A medical man who treats bony injuries obviously gains confidence and keener perception in both minor and major problems. Secondly in many cases of fracture, the broken bone may not be the major issue with regard to management. A department where skin loss, soft-tissue injury and other lesions can all receive equal attention is most desirable. Orthopaedic surgeons engaged in a busy practice cannot easily give equal consideration to all sides of a complicated case. This is, of course, not due to any lack of ability, but to a lack of time and of recent experience. An accident service with accommodation for cases in which bones are broken and soft tissues are disrupted is the ideal plan. In it there should be facilities for a surgical team which is interested in all aspects of trauma to work. Other authorities may be consulted, but there will be no division of responsibility, and the staff will be familiar with all types of treatment. In such a department there would be ample opportunity for a surgeon to carry out satisfying work, and to advance both his own and general experience.

### CASUALTY CONSULTANTS REQUIRE BALANCED WORK: ACUTE AND NON-ACUTE

The immediate treatment of any condition is closely bound up with and cannot be separated from the whole course of therapy. The medical man who examines the case in the first instance should be fully conversant with the plan for that particular lesion. It must be recognized that the senior staff of a casualty department must be able to carry out both definitive treatment and non-acute surgery. The surgeon in charge of casualty must have facilities in the department or elsewhere for non-urgent surgery. Apart from this, no-one can spend a lifetime giving opinions on acute abdominal emergencies if he can never follow up his clinical judgement by operation, nor can he be expected to remain interested in the treatment of burns in the acute phases if they always pass from his care in the very early stages. It is obvious that the casualty consultant's opinion will cease to be of much value if he ceases to be an active member of the profession. To keep his balance he must have an outlet for his training and ability and a means of gaining further experience. Therefore, it is obvious that there must either be two consultants or one consultant with a deputy of considerable experience in the casualty department in order that there should be adequate service throughout the twenty-four hours.

### THE FUTURE

Looking ahead, it would seem that our casualty departments must develop into accident units or hospitals. Then all accidents, both great and small, can be directed to the hospital where all will know that there are full and adequate facilities available. Both doctors and patients will regain lost faith and confidence and closer co-operation between the general practitioner, factory doctor, and hospital officer will lead to better treatment and less misunderstanding. Then a case can be treated by all together or each in turn, and each one will know the plan of treatment for the case and will know the aims of his colleagues although all will be working from a different angle. If Accident Centres are evolved, the hospitals will have senior staff who have an interest and work for a lifetime of service, and the doctors will have colleagues in the hospital who will share their difficulties in the accident cases, and in those cases requiring urgent opinion, not falling into the narrow channels which are available at present.

No matter what changes take place, we must all try to see that our casualty departments are kept free from unnecessary and unsuitable cases. The doctor looks to the hospitals for a better casualty service and he must get it, but the hospital looks to him to sort out the patients so that only those who will benefit from immediate treatment are sent up. The public finds the open doorway a great advantage and discouragement alone will not make it turn away. The restoration of faith in the family doctor which appears to be evident, and on the increase, will do more than an overpowering sister or a forbidding doctor. Again closer co-operation between hospitals and doctors will improve matters. The good patients suffer and the bad thrive on a system of medical planning in which one doctor does not know the other. The opportunities for the exploitation of such a system are many when communication between colleagues in different spheres is negligible or absent.

An efficient casualty department is essential, but equally important are the health centres and the surgeries. The central department and the peripheral surgery are complementary, and their closer integration must be the aim of all connected with medical administration of both hospital and general-practice services.

